# Spectrophotometric determination of sulfanilamide using PMDA as a π-acceptor: method optimization, validation, and computational study

**DOI:** 10.1039/d5ra06443k

**Published:** 2025-10-28

**Authors:** Adar Jamal Faris, Mahmood Ali Hasan, Shinwar A. Idrees

**Affiliations:** a Department of Chemistry, College of Science, University of Zakho Kurdistan Region Iraq shinwar.idrees@uoz.edu.krd

## Abstract

A new, quick, economical, accurate and precise spectrophotometric method was developed for the determination of sulfanilamide in its pure form. This technique focused on the reaction of this amine as an n-donor *via* the charge-transfer complex formation reaction with pyromellitic dianhydride (PMDA) as a π-acceptor. The maximum absorption of sulfanilamide was at 258 nm. Beer's law was observed over the concentration range of 0.2–35 μg mL^−1^ with a molar absorptivity value of 16 226.24 L mol^−1^ cm^−1^. The accuracy of the method (average recovery %) was 101.501%, and its precision (RSD %) was less than 0.6%. The nature of PMDA complexes with sulfanilamide was studied using the mole ratio method. The findings demonstrated that this complex was produced in a ratio of 1 : 1 with a stability constant of 2.49 × 10^8^ L mol^−1^ for the amine-PMDA complex. The detection limit (LOD) was 0.0136 μg mL^−1^, and the limit of quantification (LOQ) was 0.0412 μg mL^−1^. DFT calculations were employed to learn more about the charge transfer mechanism. Density of states (DOS) calculations demonstrated electronic interactions near the Fermi level, in which nitrogen and sulfur orbitals from sulfanilamide act as electron donors, while the carbonyl group and aromatic system of PMDA act as electron acceptors. HOMO–LUMO analysis further confirmed this donor–acceptor mechanism.

## Abstract 2

A new, quick, economical, accurate and precise spectrophotometric method was developed for the determination of sulfanilamide in its pure form.

## Graphical abstract

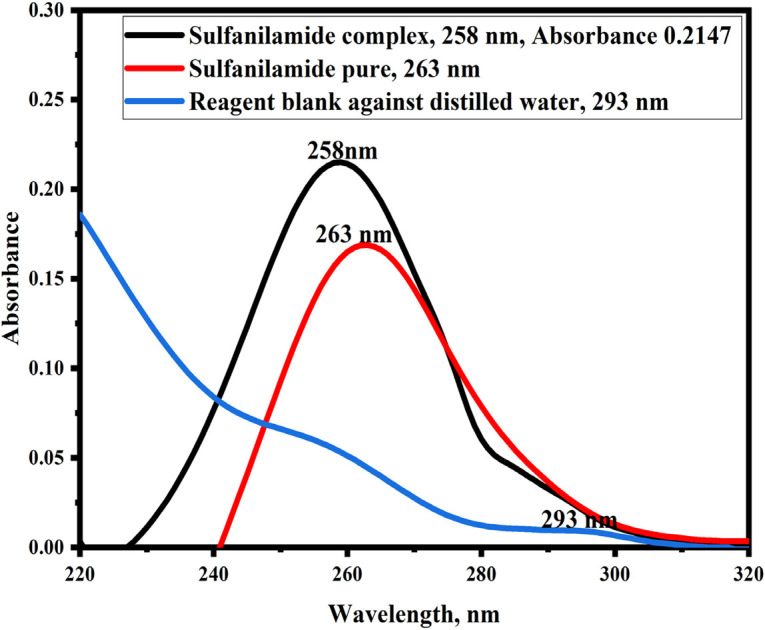

## 1. Introduction

Usually, a new distinctive absorption band can be observed when two molecules are mixed together, which are likely to form loose complexes. This new absorption band appears due to the transition of an electron from the high charge density of the molecule (known as the donor) to the lower charge density of the molecule (known as the acceptor). The donor (D)-to-acceptor (A) transition of the electron occurs from the HOMO (highest occupied molecular orbital) to the LUMO (lowest unoccupied molecular orbital) of the donor and acceptor, respectively. The additional CT (charge transfer) absorption band is due to this transfer of the electron from the donor to the higher unoccupied orbital of the acceptor.^1–3^

Sulfanilamide, chemically known as 4-aminobenzenesulfonamide, is a medical compound used to protect against certain bacterial illnesses. It is frequently used in the form of a topical cream or powder to treat surface diseases and in the form of a pill for internal illnesses. It falls into the category of sulfonamide antibacterial drugs. Common diseases cured by sulfanilamide include urinary tract infections, vaginal infections, strep throat, and some staph infections^4^–.infections, strep throat, and some staph infections.^4–7^

Different analytical methods have been reported for the determination of sulfanilamide as an organic amine compound. These methods include high-performance liquid chromatography,^8^ electrochemical detection^9^ and colorimetric techniques.^10^ Most of these methods are time-consuming, expensive or tedious, requiring extraction steps or highly sophisticated instrumentation. Charge-transfer methods using π-acceptors such as DDQ and TCNE are well documented; the application of PMDA specifically for sulfanilamide determination has not yet been reported. The comparison between different methods illustrated in Table 1 strengthens the context. The emphasis on PMDA offers advantages over other π-acceptors, which include higher stability of the 1 : 1 complex, excellent aqueous solubility (avoiding organic solvents), and lower reagent costs. These features make the method more accessible, rapid, and eco-friendly, extending its potential for broader application in quality control and routine analysis. The present work is a development of a new, straightforward, accurate and precise spectrophotometric method for the determination of sulfanilamide in its pure form. The method is based on the charge-transfer complex formation reaction of the above amine compounds as n-donors with pyromellitic dianhydride reagent as a π-acceptor, without requiring any derivatization or catalysis.

**Table 1:** Comparison of the reported analytical methods for sulfanilamide determination

Method	LOD (μg mL^−1^)	Linear range (μg mL^−1^)	Analysis time	Cost/solvent/practicality	Ref.
HPLC	0.04–0.06	0.01–1000	Long (sample extraction/cleanup + chromatographic run; typical runs last tens of minutes)	High instrumentation and operating cost; very high sensitivity and selectivity	17
Electrochemical (square-wave voltammetry)	0.92 μmol L^−1^	∼0.86–12.9	Fast measurement (seconds–minutes) after sensor preparation; sensor fabrication time may apply	Low-cost portable instrumentation possible; excellent LOD in many sensor designs	18
Spectrophotometric determination (1,2-naphthoquinone-4-sulfonic acid@sulfonamides) condensation reaction method	0.546–0.536	5–30	Fast (minutes); often requires small amounts of organic reagents	Low instrumentation cost; some methods require organic solvents or derivatization	19
Charge-transfer using π-acceptors [2,3-dichloro-5,6-dicyano-1,4-benzoquinone (DDQ) and tetracyanoethylene (TCNE)]	Typical CT spectrophotometric LODs reported across sulfanilamide concentrations: low μg mL^−1^ depending on reagent and solvent; values are reagent- and setup-specific	Often sub-μg mL^−1^ → tens of μg mL^−1^ (method dependent)	Quick (in minutes) once the reaction is established; a lot of CT reagents are utilized in organic media	DDQ/TCNE gives very intense CT bands (high sensitivity) but often requires organic solvents and/or more expensive reagents than PMDA.	20
PMDA charge-transfer (present manuscript)	0.0136	0.2–35	Quick formation; improvement time of 5 min, stability of ∼20 min (aqueous)	Economical reagent; aqueous medium; no extraction; basic UV-vis equipment	—

The combined experimental and theoretical study provides information that is difficult to obtain experimentally. Spectrophotometry, particularly UV-vis spectroscopy, is widely used for drug quantification due to its simplicity, cost-effectiveness, and rapid analysis. Theoretical calculations, specifically DFT calculations, complement experimental understanding by providing theoretical insights into molecular geometry, electronic transitions, and absorption spectra. As a result, researchers can predict absorption spectra before and after interactions, followed by interpreting spectral behaviors. Additionally, DFT helps to predict intermolecular interactions, charge transfer, solvent effects, and the influence of pH on drug absorbance. This combined approach offers insights into drug–reagent interactions, stability, and reactivity. Furthermore, DFT-based molecular orbital analysis, *e.g.*, HOMO–LUMO and energy gap, can explain the reactivity, stability, and mechanism pathways of drug–reagent interactions.^11–16^

In this research, a novel, rapid, low-cost, accurate, and precise spectrophotometric method was developed for the determination of sulfanilamide in its pure form. The method is based on the formation of a charge transfer (CT) complex between sulfanilamide@PMDA, which serves as a π-acceptor reagent. In addition, DFT calculations were employed to gain deeper insights about the charge transfer mechanism.

## 2. Experimental section

### 2.1. Apparatus

All spectrophotometric measurements were performed using a PerkinElmer, Lambda 25 UV-visible double beam spectrophotometer with matching 1 cm quartz cells. Weighing was carried out using an A&D (GR-200) balance, which is a sensitive balance, with four-digit precision. Heating of the solutions was carried out on a GFL 1003 water bath (Germany). The pH measurements were made by using a Mettler Toledo FE20-Basic pH meter with its combined glass electrode.

### 2.2. Reagents

All of the compounds used were of the highest quality available. Sodium hydroxide was purchased from Alfa Aesar (Germany). Potassium hydroxide and carbonate were obtained from Alpha Chemika (India). Sulfanilamide, dimethyl sulfoxide, Tween-80 and ammonia solution were purchased from Merck (Germany). Acetone and phosphate were supplied by Roth (Germany). Methanol was acquired from Thomas Baker (India). Hydrochloric acid and ethanol were obtained from Scharlau (Spain). Cetyltrimethylammonium bromide and sodium dodecyl sulfate were obtained from Sigma-Aldrich (Germany). Acetonitrile was purchased from BDH (England).

Pyromellitic dianhydride solution (1 × 10^−3^ mol L^−1^) was freshly prepared by dissolving 0.0218 g of PMDA in distilled water and diluting it to the mark in a 100 mL volumetric flask. This solution was prepared daily and used immediately.

Standard solutions of sulfanilamide (100 μg mL^−1^) were freshly prepared by dissolving 0.01 g of sulfanilamide in distilled water and diluting it to the mark with distilled water in a 100 mL volumetric flask. The solution was prepared daily and used immediately.

Surfactant solutions (0.1%) were prepared by dissolving 0.1 g of each surfactant—(positive) cetyltrimethylammonium bromide (CTAB), (negative) sodium dodecyl sulfate (SDS) and (neutral) Tween-80—in 100 mL of distilled water.

### 2.3. General procedure

Aliquots of standard solutions of sulfanilamide were placed into a series of 10 mL volumetric flasks. After that, 0.1 mL of 1 × 10^−3^ mol L^−1^ PMDA reagent for sulfanilamide was added, and the contents were mixed well and diluted to the mark with distilled water. The absorbance at *λ*_max_ was measured against a reagent blank.

### 2.4. Computational simulation

To gain more knowledge about the work and mechanism of CT between sulfonamide and PMDA, computational simulations were adopted in the context of DFT calculations using the local code GGA/PBE functional. The calculations were carried out using the Dmole^3^ code in Material Studio version 2020. In these calculations, the plane wave basis set and the basis set selected were Double Numerical plus *d*-functions (DND) with a polarization d-function on all non-hydrogen atoms. SCF tolerance was 1 × 10^−5^ eV per atom, and the smearing was determined to be 0.005 Ha. Molecular Dynamics (MD) simulations were also performed to acquire more details about the adsorption energy between the sulfonamide and the PMDA molecule. The calculations were performed using universal force field integration combined with the MD method.

## 3. Results and discussion

### 3.1. Absorption spectra

**Fig. 1:**
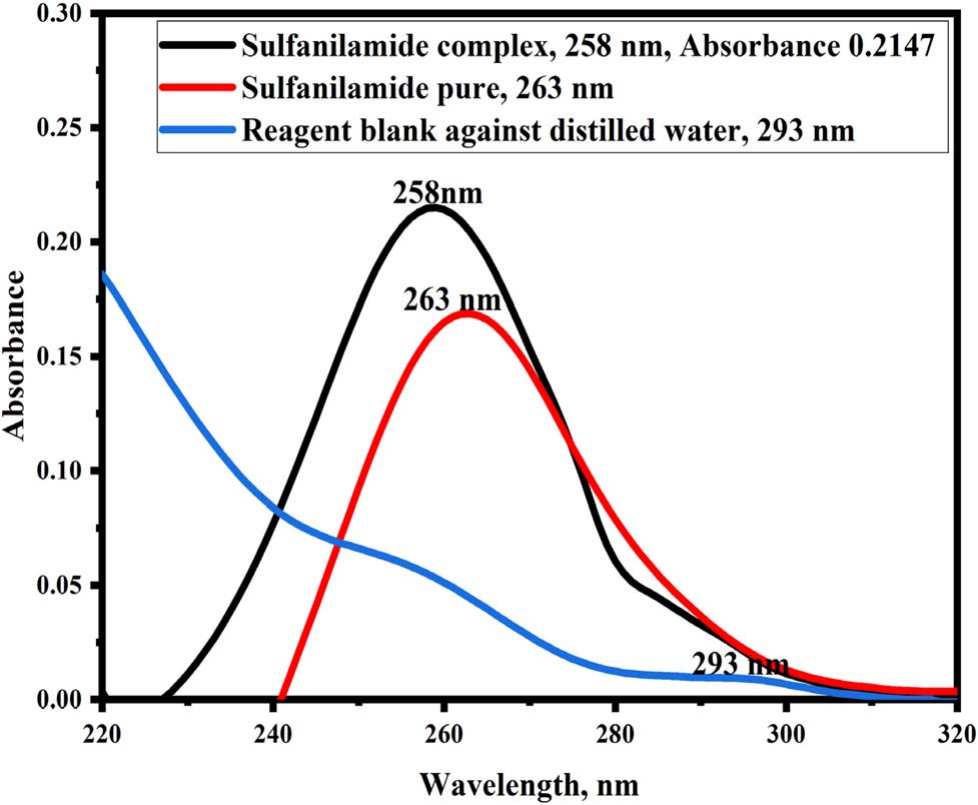
Absorption spectra of 2 μg mL^−1^ sulfanilamide *versus* reagent blank.

Sulfanilamide forms a charge transfer complex with the PMDA reagent, having maximum absorption at 258 nm. Fig. 1 shows the absorption spectrum of sulfanilamide@PMDA, showing a red shift to 293 nm. A hyperchromic shift was also noticed at 258 nm in the sulfanilamide@PMDA spectra. As a result, the wavelength at 258 nm was selected as *λ*_max_ in all subsequent measurements.

### 3.2. Optimizations

Various experimental parameters that influence the reaction development and its stability were carefully examined and optimized. These parameters included the effects of solvent, pH and buffer solution, reagent concentration, order of addition, temperature, developing time and surfactants.

#### 3.2.1. Effect of solvent

Different solvents were examined, such as distilled water, ethanol, methanol, acetone, acetonitrile and dimethyl sulfoxide, for the study of the sulfanilamide@PMDA complex to attain the highest sensitivity and product stability. It was noticed that using distilled water as a solvent for the PMDA reagent, sulfanilamide and sulfanilamide@PMDA gives maximum absorbance intensity in both concentrated and diluted forms. Therefore, distilled water was selected as a solvent in all subsequent experiments.

#### 3.2.2. Effect of pH and buffer solution

**Fig. 2:**
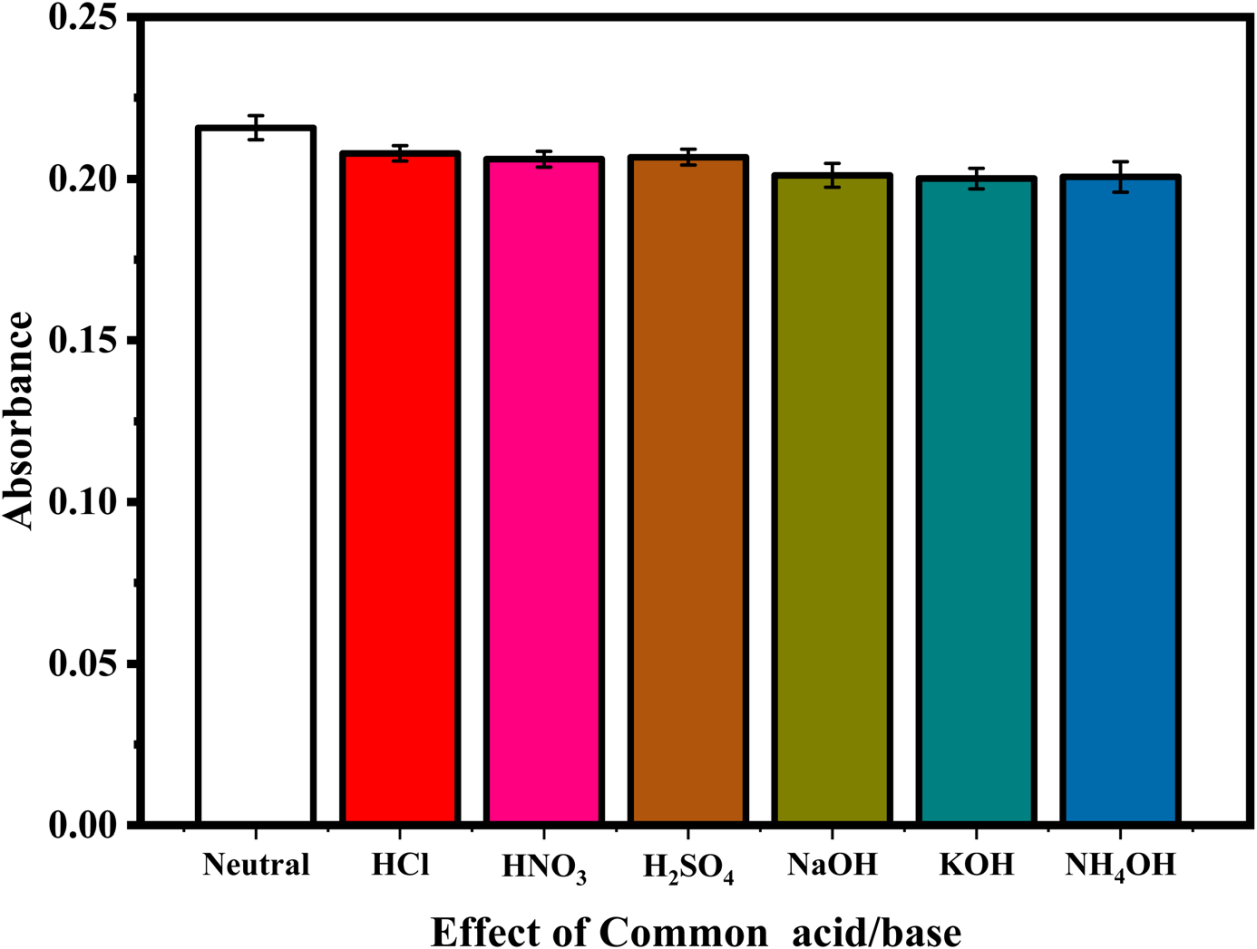
Effect of different pH and common acids and bases on the absorbance of sulfanilamide.

The influence of different acids and bases on the intensity was investigated using 0.1 mol L^−1^ of HCl, HNO_3,_ H_2_SO_4_, NaOH, KOH and NH_4_OH. The results indicate that the sulfanilamide@PMDA complex was not affected by adding acids or bases, demonstrating the robustness of the method. The effects of different acids and bases are shown in Fig. 2.

#### 3.2.3. Effect of PMDA reagent concentration

**Fig. 3:**
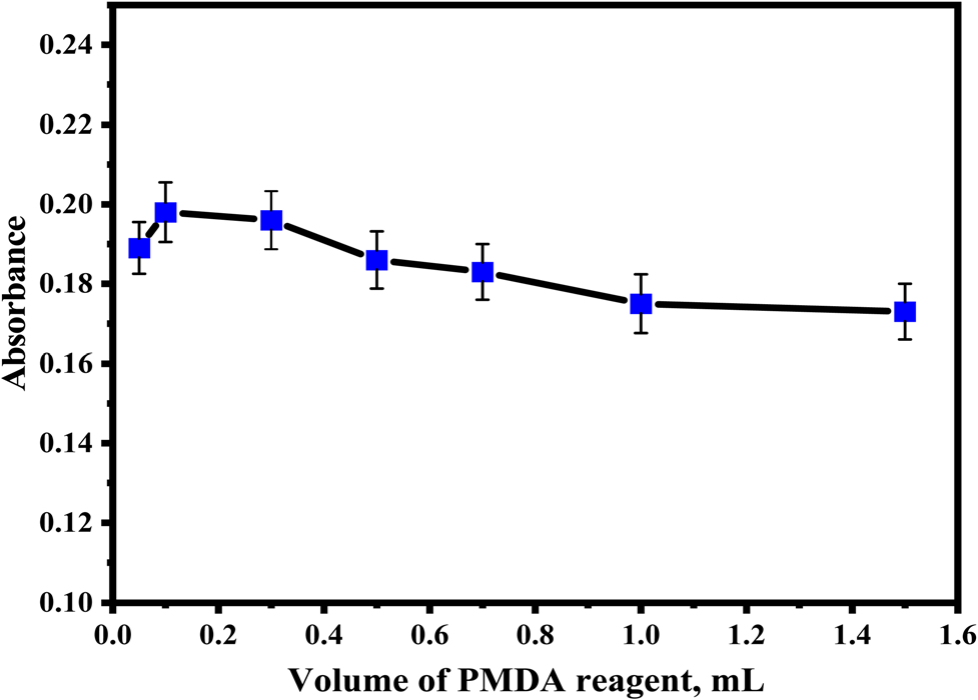
Effect of different volumes of the PMDA reagent on the absorbance of sulfanilamide.

In order to study the effect of the concentration of the reagent on the absorbance of the charge transfer complexes, different volumes of 1 × 10^−3^ mol L^−1^ PMDA reagent were added to a solution containing a constant amount of sulfanilamide. It was observed that the absorbance increased with increasing reagent concentration and reached a maximum value when using a volume of 0.1 mL PMDA reagent. Fig. 3 shows the effect of the addition of different PMDA volumes.

#### 3.2.4. Effect of reaction time and temperature

**Fig. 4:**
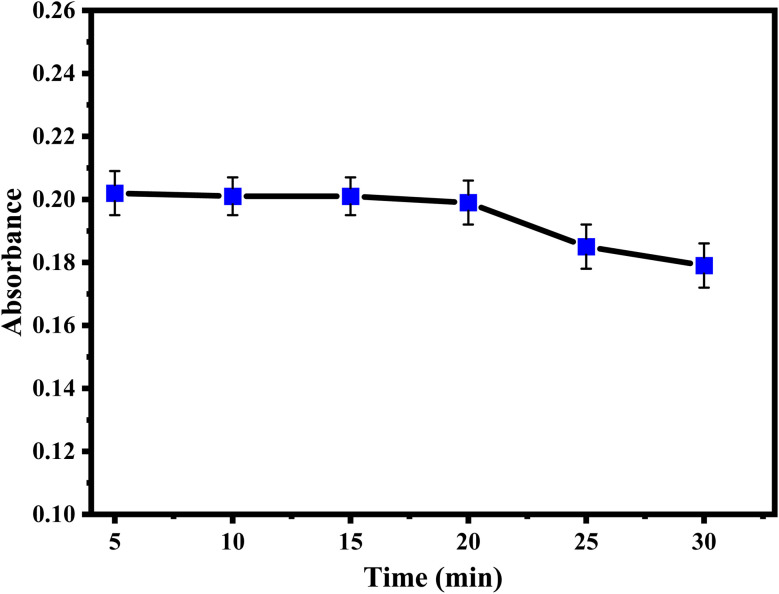
Effect of temperature and developing time on the absorbance of sulfanilamide.

The impacts of temperature and reaction time were studied using a thermostatically controlled water bath at different temperatures ranging from 20 °C to 45 °C and different time periods. Regarding the temperature effect, the results show that the sulfanilamide@PMDA complex was formed immediately after the addition of the reagent and reached its maximum absorbance at 30 °C. As for the stability of sulfanilamide@PMDA over a period of time, it remained stable for 20 minutes, as illustrated in Fig. 4.

#### 3.2.5. Effect of the order of addition

The influence of the order of addition on the absorbance of the sulfanilamide@PMDA complex was investigated. Maximum sensitivity was achieved when the PMDA reagent was added first to sulfanilamide. This order was followed in the subsequent procedures.

#### 3.2.6. Effect of surfactants

The influence of different surfactants, such as the positive surfactant cetyltrimethylammonium bromide, the negative surfactant sodium dodecyl sulfate, and the neutral surfactant Tween-80, was studied. Increasing the volume of CTAB, SDS, or Tween-80 (0.2–1 mL) caused a gradual decrease in absorbance compared with the control (no surfactant). This trend demonstrates that the negative influence of surfactants was consistent across different added volumes. These results strengthen the robustness of our conclusions by confirming that the observed effect was not limited to a single experimental condition but persists across a range of surfactant volumes, as shown in Table 2.

**Table 2:** Effect of surfactants on the amine-PMDA complexes

Surfactant added (mL, 0.1%)	Absorbance		
	CTAB	SDS	Tween-80
Without	0.214	0.214	0.214
0.2 mL	0.202	0.204	0.196
0.5 mL	0.203	0.205	0.198
0.7 mL	0.207	0.208	0.201
1 mL	0.201	0.209	0.199

#### 3.2.7. Optimum reaction conditions

The optimum reaction conditions for sulfanilamide are summarized in Table 3.

**Table 3:** Optimum reaction conditions for the determination of amines

Compound	*λ*_max_ (nm)	Final pH	Temp. (°C)	PMDA (1 × 10^−3^ mol L^−1^)	NaOH (0.1 mol L^−1^)	Development time (min)	Stability period (min)
Sulfanilamide	258	3.84	30	0.1	0 mL	5	20

#### 3.2.8. Method validation

**Fig. 5:**
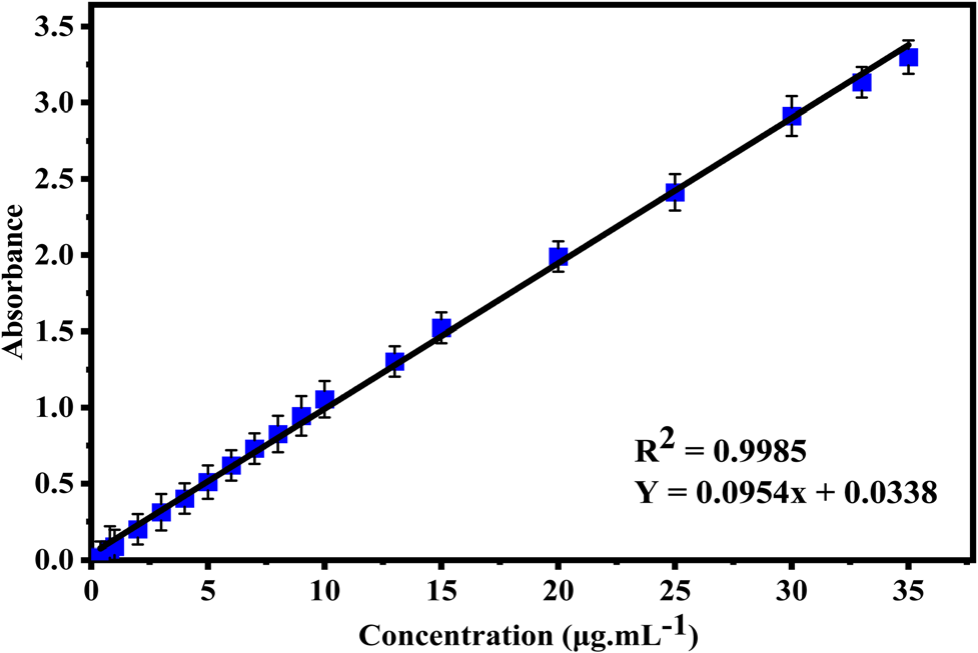
Calibration curve for the determination of sulfanilamide.

Method validation was also studied in order to explore the range in which sulfanilamide@PMDA adheres to the Beer's law. The absorbance was plotted *versus* concentration (μg mL^−1^), as shown in Fig. 5. The regression equation demonstrates linearity, and the suggested method yielded a coefficient of determination greater than 0.99, indicating excellent linearity. The limits of the Beer's law, molar absorptivity, and Sandell's sensitivity were assessed and tabulated in Table 4.

**Table 4:** Overview of the optical properties and data for the suggested technique

Parameter	Sulfanilamide
*λ*_max_ (nm)	258
Limits of the Beer's law (μg mL^−1^)	0.2–35
Molar absorptivity (L mol^−1^ cm^−1^)	16 226.24
LOD (μg mL^−1^)	0.0136
LOQ (μg mL^−1^)	0.0412
Sandell's sensitivity (μg cm^−2^)	0.0104
Coefficient of determination (*r*^2^)	0.9985
Slope	0.0954
Intercept	0.0338

#### 3.2.9. Accuracy and precision

The accuracy and precision of the suggested method were tested by measuring the content of intended sulfanilamide@PMDA at three different concentration levels (low, medium and high) by measuring five replicates of sulfanilamide at 3, 10 and 25 μg mL^−1^, as illustrated in Table 5. Precision was estimated through the relative standard deviation (RSD%), whilst the mean recovery represented the accuracy. The RSD% values were less than 0.6%. These results demonstrate that the proposed method was accurate and precise.

**Table 5:** Accuracy and precision of the proposed method

Compound	Amount added (μg mL^−1^)	Amount found (μg mL^−1^)	Recovery^a^ (%)	Average recovery (%)	RSD^a^ (%)
Sulfanilamide	3	2.988	99.600 ± 0.00023	101.501	0.074
	10	10.436	104.360 ± 0.00529		0.514
	25	25.136	100.544 ± 0.00243		0.100

a Average of five determinations.

#### 3.2.10. Stoichiometry and stability constant

**Fig. 6:**
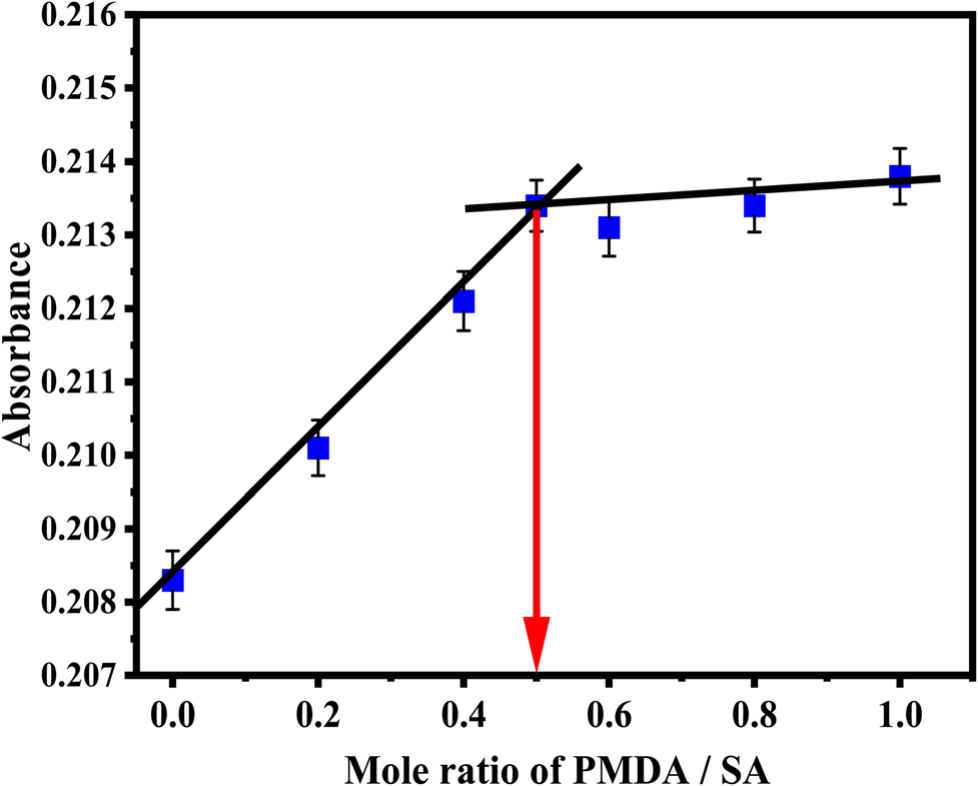
Molar ratio plots for sulfanilamide@PMDA products.

The stoichiometry of the reaction of the sulfanilamide@PMDA complex was also examined utilizing the mole ratio method. A molar ratio plot was drawn by adding different volumes (0.2–1 mL) of 2.5 × 10^−4^ mol L^−1^ PMDA to a constant amount (0.5 mL of 2.5 × 10^−4^ mol L^−1^) of sulfanilamide solution. The suggested method was applied under the optimum conditions, and the absorbance was measured at the corresponding wavelength. The result of the mole ratio method is presented in Fig. 6. The results indicate that the sulfanilamide@PMDA complex was formed in the ratio of 1 : 1. This reveals that the amine group present in sulfanilamide was responsible for the formation of the charge-transfer complex.

The apparent stability constant was calculated by comparing the absorbance of a solution containing stoichiometric amounts of sulfanilamide@PMDA (*A*_s_). The complex was appreciably dissociated, and as expected, it showed lower absorbance than the one containing an excessive amount of the PMDA reagent (*A*_m_), in which the sulfanilamide@PMDA complex had been largely associated and consequently had a greater absorbance. The difference between the two absorbance values provides a measure of the complex's degree of dissociation (α) as follows:1
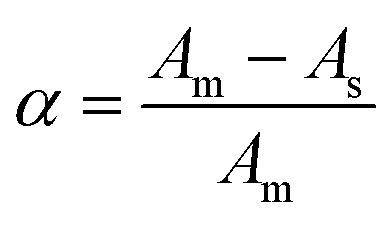


On the basis of the previous experiment, if a donor–acceptor complex is formed by the interaction of the donor component D (amine) and acceptor component A (PMDA), it follows eqn (2) and (3):2
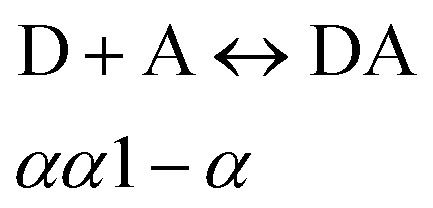


Then, the stability constant can be written as follows:3
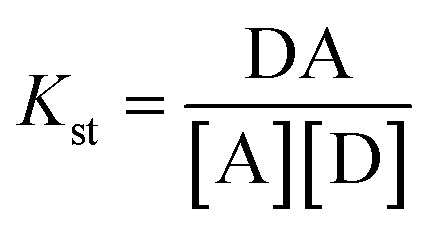


If *α* is the degree of dissociation and *C* is the concentration of the complex, then eqn (4) can be written as follows:4
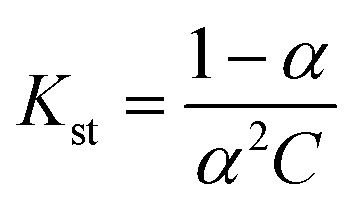
where *K*_st_ is the stability constant of the complex, *C* is the final molar concentration of sulfanilamide, and *α* is the degree of dissociation. The results obtained from the determination of *K*_st_ for sulfanilamide are given in Table 6, where the results indicate that the sulfanilamide@PMDA complex is stable.

**Table 6:** Stability constant of amine-PMDA complexes

Compound	Conc. (mol L^−1^)	Absorbance		α	*K*_st_ (L mol^−1^)
		*A*_s_	*A*_m_		
Sulfanilamide	0.00001	0.1435	0.1485	0.03367	2.49 × 10^8^
	0.000015	0.212	0.2169	0.02259	
	0.00002	0.2901	0.2971	0.02356	
	0.000025	0.3681	0.3709	0.3709	

#### 3.2.11. Selectivity

**Fig. 7:**
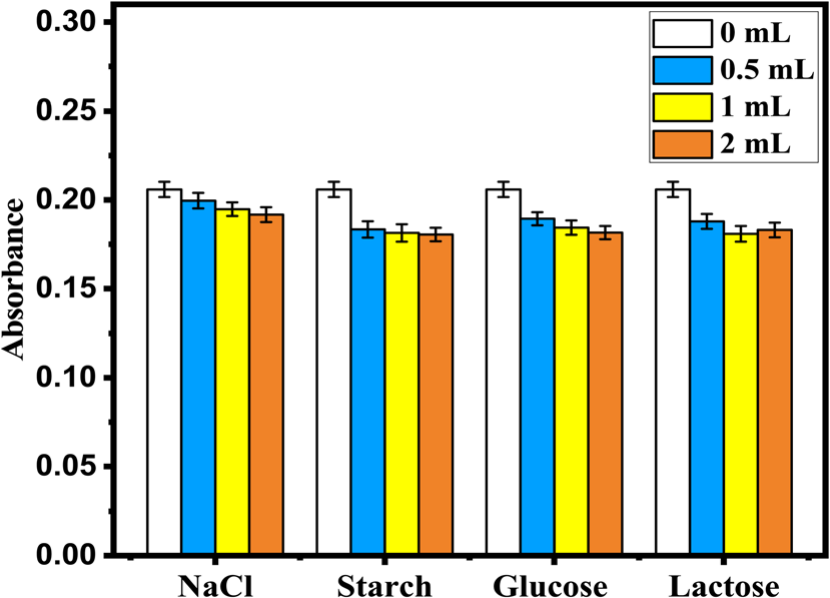
Effect of common excipients (NaCl, starch, glucose and lactose) on the determination of the sulfanilamide-PMDA complex.

The proposed method was found to be selective for the determination of sulfanilamide in the presence of various excipients such as NaCl, starch, glucose and lactose (Fig. 7). It was found that the studied excipients did not interfere with the determination of sulfanilamide.

### 3.3. Theoretical study

**Fig. 8:**
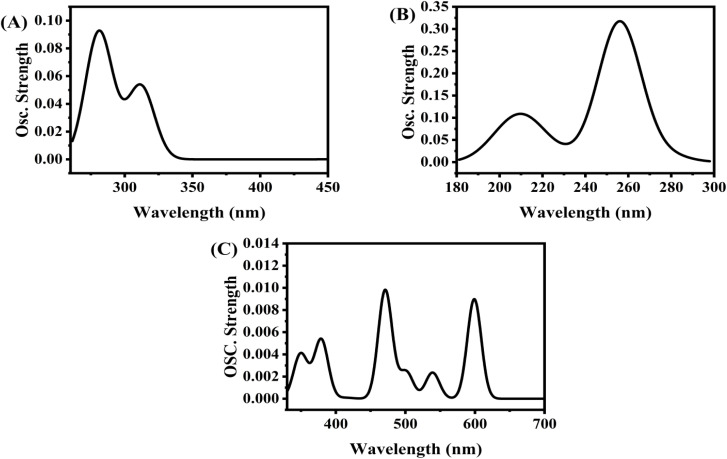
TD-DFT excitations as a function of oscillator strength of (A) PMDA, (B) sulfonamide and (C) sulfonamide@PMDA by using GGA/BEP/DND.

To have a better understanding of the mechanism of charge transfer between sulfonamide and PMDA, computational simulations were performed in the context of DFT calculations using the local code GGA/PBE functional. Fig. 8 and Table 7 show theoretical UV-vis spectra and their calculated parameters, which illustrate the spectrophotometric determination of sulfonamide using PMDA as a derivatizing agent. Fig. 8A shows the PMDA spectrum, which shows absorption in the UV-vis range, while Fig. 8B presents the spectrum of sulfonamide, which shows strong absorption peaks in the far UV area. As presented in Fig. 8C, the sulfonamide@PMDA complex displays a red shift into the visible region (400–700 nm), with weaker oscillator strength but being more detectable at longer wavelengths in the visible region. This shift confirms the formation of a charge-transfer (CT) complex, enabling selective and sensitive sulfonamide quantification. The proposed method holds PMDA's ability to form a complex with sulfonamide, transforming its UV-dominant absorption into a visible-range signal.^11–16^

**Table 7:** TD-DFT optic properties of PMDA, sulfonamide and sulfonamide@PMDA

Parameter	PMDA	Sulfonamide	Sulfonamide@PMDA
Wavelength range	300–450 nm (UV-vis)	180–300 nm (far-UV/UV)	Likely 400–700 nm (visible, red-shifted)
Peak osc. Strength	∼0.08 (350 nm)	∼0.35 (200–240 nm)	∼0.014
Transition type	π → π/n → π (conjugation)	Π → π* (aromatic/heteroatom)	Charge-transfer (CT) complex

**Fig. 9:**
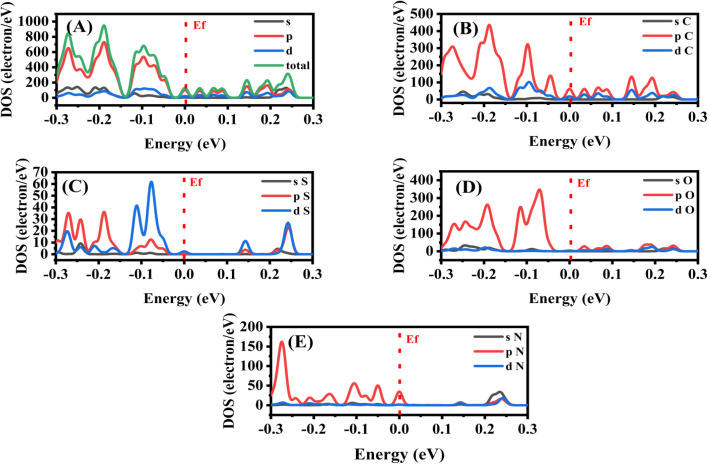
Density of states (DOS) of sulfonamide@PMDA, (A) TDOS, (B) DOS of the carbon atom, (C) DOS of the sulfur atom, (D) DOS of the oxygen atom, and (E) DOS of the nitrogen atom by using GGA/BEP/DND.

Fig. 9 shows the Density of States (DOS) analysis of the sulfonamide@PMDA complex. The total DOS, shown in Fig. 9A, exhibits significant electronic states near the Fermi level (*E*_f_), indicating strong interactions between sulfonamide and PMDA. For the carbon atoms primarily from PMDA, as presented in Fig. 9B, the p-orbitals dominate near *E*_f_, reflecting the π-conjugated system's role in electron delocalization. Fig. 9C shows PDOS for sulfur atoms from the sulfonamide –SO_2_ group, which exhibits d-orbital contributions at *E*_f_, which suggest the involvement of the –SO_2_ group in charge transfer. Fig. 9D presents PDOS for oxygen, which represents both the carbonyl group from PMDA and the sulfonyl group from sulfonamide. It shows broad p-orbital peaks at the valence band near *E*_f_, which confirms the electron-accepting capability. Fig. 9E illustrates PDOS for nitrogen p-orbital states, which is derived from the NH^−^ group of sulfonamide, which highlights the electron-donating character. All in all, these DOS show how nitrogen and sulfur from sulfonamide give electrons to the carbon and oxygen system of PMDA, resulting in the formation of a stable charge-transfer complex.

**Fig. 10:**
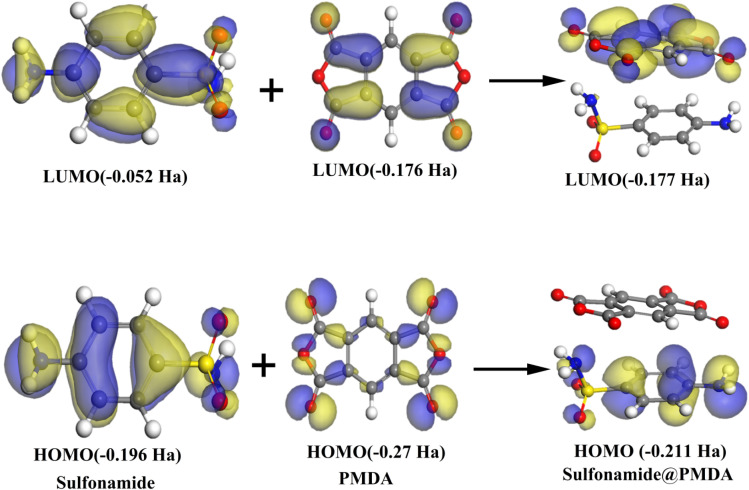
Highest occupied molecular orbitals (HOMO) and lowest unoccupied molecular orbital (LUMO) of sulfonamide, PMDA, and sulfonamide@PMDA by using GGA/BEP/DND.

Fig. 10 shows HOMO–LUMO analysis of sulfonamide, PMDA, and their interaction to form the sulfonamide@PMDA complex. Regarding sulfonamide, the HOMO is localized on the nitrogen lone pair and the aromatic ring. As a result, it can be identified as an electron donor, while the relatively high energy of the LUMO (−0.052 Ha) indicates poor electron acceptance. On the other hand, PMDA exhibits a low energy of the HOMO (−0.27 Ha) delocalized across its carbonyl groups and benzene ring, confirming its electron-withdrawing character. When these components form the sulfonamide@PMDA complex, the HOMO at −0.211 Ha energy remains primarily localized on the sulfonamide part, while the LUMO at −0.177 Ha energy closely represents PMDA orbitals, demonstrating that charge transfer occurs from the HOMO of sulfonamide to the LUMO of PMDA. This interaction reduces the energy gap in the complex compared with isolated sulfonamide and PMDA, which explains the observed red shift in the UV-vis spectra resulting from lower-energy electronic transitions. These computational findings agree well with the experimental UV-vis data, which provide a comprehensive understanding of the electronic structure of the complex formation and behavior.^11–16^

All in all, the sulfonamide@PMDA complex undergoes charge transfer transitions, which mostly occur between the electron-donating group of sulfonamide and the electron-accepting group of PMDA. In sulfonamide, the nitrogen lone pair electrons from the –SO_2_NH– group and the aromatic π-electrons act as the electron donors, while in PMDA, the electron-deficient carbonyl groups (C=O) of the anhydride functional and the conjugated π-system of the benzene ring serve as the electron acceptors. This donor and acceptor interaction facilitates a charge transfer transition, where electrons from sulfonamide are partially transferred to PMDA, resulting in a new absorption band at longer wavelengths. The observed red shift in the sulfonamide@PMDA spectrum, as presented in Fig. 2C, compared to the spectra of PMDA and sulfonamide separately, as shown in Fig. 2A and B, shows the characteristic of such CT complexes, as the energy required for electronic transitions decreases due to the delocalization of charge. The weaker oscillator strength in sulfonamide@PMDA supports the CT nature of the transition, which involves symmetry forbidden or partially forbidden states. Finally, based on the MD results, it can be inferred that sulfonamide adsorption with PMDA requires a negative adsorption energy (−160.449 eV) and is therefore energetically favorable.

## 4. Conclusions

A new, sensitive, economical, accurate and precise spectrophotometric method has been developed for the determination of sulfanilamide. The method demonstrates excellent precision, linearity, and reproducibility, with low detection limits and straightforward aqueous operation. The method does not require various elaborate treatments and tedious extraction procedures, and it involved basic instrumentation, showing that the method is straightforward.

With regard to the theoretical study, it provides fundamental insights into the charge-transfer (CT) interactions between sulfonamide and PMDA, which elucidate the electronic and optical properties of the sulfonamide@PMDA complex. The UV-vis spectral analysis demonstrated a significant red shift, confirming the formation of a stable CT complex with enhanced absorption in the visible region. DOS calculations also showed a strong electronic coupling near the Fermi level, with nitrogen and sulfur orbitals from sulfonamide and the carbonyl group and π-conjugated systems from PMDA. The HOMO and LUMO analysis further validated the donor–acceptor mechanism, which shows the charge transfer from sulfonamide HOMO to PMDA LUMO with a reduced energy gap that explains the unique photophysical behavior of the sulfonamide@PMDA complex.

## Conflicts of interest

The authors declare there are no conflicts to declare.

## Data Availability

All data supporting the findings of this study are fully available without restriction.
